# Examining the Presence of *Cronobacter* spp. in Ready-to-eat Edible Insects

**DOI:** 10.14252/foodsafetyfscj.D-19-00004

**Published:** 2019-09-24

**Authors:** Jake P. Greenhalgh, Daniel Amund

**Affiliations:** School of Life Sciences, Faculty of Health and Life Sciences, Coventry University, Priory Street, Coventry CV1 5FB, United Kingdom

**Keywords:** *Cronobacter*, entomophagy, food safety, PCR, RTE foods

## Abstract

Edible insects present a potential solution to increasing global food insecurity. However, there is limited research on the microbial hazards they may pose. These include opportunistic pathogens like *Cronobacter* spp. (formerly *Enterobacter sakazakii*). In this study, nine types of ready-to-eat edible insect products purchased in the UK were examined for their microbial load (total aerobic count, total *Enterobacteriaceae* count), and screened for the presence of *Cronobacter sakazakii(C. sakazakii)* by selective enrichment and plating on chromogenic agar. While microbial load was generally low, presumptive *Cronobacter* spp. were detected in five of the edible insect products. Four of the isolates were identified as *C. sakazakii*, using the Remel RapID ONE biochemical test kit. Genotypic characterisation of the isolates by ITS-PCR, however, demonstrated that the isolates may be other species of *Cronobacter* instead. Further studies into understanding microbial hazards linked to edible insects for human consumption are required.

## Introduction

The consumption of insects (entomophagy) is considered as a potential solution to the increasing issues of food insecurity and malnutrition. Many insect species have been consumed worldwide, and have been shown to have protein and nutrient profiles comparable to meat^1^^)^. However, consumer attitudes may present a barrier toward the acceptance of edible insects in Western countries^2^^)^. There may also be microbial hazards linked to such edible insect products^3^^,^^4^^)^.

*Cronobacter* spp. (formerly *Enterobacter sakazakii*) are Gram negative, oxidase negative, rod-shaped bacteria, which are members of the *Enterobacteriaceae* family. They are emerging opportunistic pathogens which can cause infections in adults and infants, including necrotising enterocolitis, bacteraemia, and meningitis^5^^)^. The genus consists of seven species, most of which can cause human disease. *Cronobacter sakazakii (C. sakazakii)* is commonly associated with infant infections, while *Cronobacter malonaticus* is commonly linked to infections in adults, especially the elderly and immunocompromised^6^^)^.

*Cronobacter* spp. are ubiquitous and have been isolated from a wide range of foods, such as vegetables, herbs, spices, meat products and ready-to-eat foods^7^^)^. The presence of *C. sakazakii* in powdered infant formula is of major concern, due to its implications for infant health, and has been widely studied^8^^)^.

*Cronobacter* spp. have also been isolated from the guts of insects such as fruit flies and stable flies, which may be considered as sources of environmental contamination in foods^9^^)^. Studies involving culture-independent metagenomic analysis on edible insects suggest that *Cronobacter* may be associated with the natural microbiota of mealworms^10^^,^^11^^)^.

Marketed edible insects may be whole insects, commonly processed by blanching, followed by drying^12^^)^, or powdered^3^^)^. These are considered to be ready-to-eat. To our knowledge, no studies have examined processed edible insect products for the presence of *Cronobacter* spp. Therefore, this study aimed to examine the microbial load of ready-to-eat edible insects purchased in the UK, and in particular, determine the presence of *C. sakazakii*, using culture-based methods. We also comparatively examined the use of phenotypic and genotypic methods in identifying and differentiating presumptive *Cronobacter* isolates.

## Materials and Methods

### Sample Collection

A total of nine edible insect products (Table 1) were purchased from an online UK-based retailer (two samples of each). All insects contained no additives or additional flavouring (except for the queen leafcutter ants which were lightly salted), and were stored at room temperature.

**Table 1.** Ready-to-eat insects used in this study, including country of origin, method of processing and bacterial counts (log CFU/g)

**Insect**	**Latin Name**	**Country of Origin**	**Processing Method**	**Total Aerobic Count**	***Enterobacteriaceae***
Buffalo Worms	*Alphitobius diaperinus*	Netherlands	Freeze-dried	4.59	< 2.00
Crickets	*Acheta domesticus*	Netherlands	Freeze-dried	3.95	< 2.00
Cricket Flour	*Acheta domesticus*	Thailand	Dehydrated, finely milled	4.00	2.57
Giant Waterbugs	*Lethocerus indicus*	Thailand	Dehydrated	2.24	< 2.00
Locust	*Locusta migratoria*	Netherlands	Freeze-dried	3.72	< 2.00
Mealworms	*Tenebrio molitor*	Netherlands	Freeze-dried	2.17	< 2.00
Queen Leafcutter Ants	*Atta laevigata*	Colombia	Brine boiled and air-dried	3.35	< 2.00
Silkworm Pupae	*Bombyx mori*	Thailand	Pressure steam cooked then dehydrated	2.00	< 2.00
Wild Black Ants	*Lasius niger*	Thailand	Pressure steam cooked then dehydrated	2.24	< 2.00

### Reference Strains

*C. sakazakii* reference strains NCIMB 8272 and NCIMB 5920 were kindly provided by London Metropolitan University. Strains were confirmed by 16S rDNA sequencing^13^^)^.

### Microbiological Enumeration

A 10 g sample of each insect product was homogenized in 90 ml of buffered peptone water (BPW) (Oxoid CM1049) using a stomacher (Stomacher 400 Circulator, Seward) at 300 rpm for 1 minute. From the homogenized suspension, further serial dilutions were prepared up to 10^−4^ in BPW. Subsequently, 0.1 ml of each dilution was plated in duplicate onto nutrient agar (Oxoid CM0003) to determine total aerobic count, and violet red bile glucose (VRBG) agar (Oxoid CM1082) to determine total *Enterobacteriaceae* count. The inoculated plates were incubated at 37°C for 24 h. All enumeration experiments were carried out in two replicates.

### Detection and Isolation of Presumptive *Cronobacter* spp*.*

To detect *Cronobacter* spp., the initial homogenized suspension of each insect sample in BPW, as made previously for enumeration, was incubated at 37°C for 24 h (pre-enrichment). Afterwards, 0.1 ml of the pre-enrichment was suspended in 10 ml of *Cronobacter* Screening Broth (CSB) (Sigma-Aldrich 38948) supplemented with vancomycin (Sigma-Aldrich 75423) according to manufacturer’s instructions, and incubated for 24-48 h at 37°C. If the CSB changed color from purple to yellow, then the sample was considered positive for the presence of *Cronobacter* spp. due to fermentation of sucrose^14^^)^. A loopful of each positive broth was then inoculated onto chromogenic medium HiCrome *Cronobacter* spp. Agar (Sigma-Aldrich 92324) and incubated for 24 h at 37°C. Any presumptive *Cronobacter* spp. would grow as dark blue colonies on this agar, due to cleaving of the chromogenic substrate by α-glucosidase, which is produced by *Cronobacter* spp.^15^^)^.

### Phenotypic and Biochemical Characterisation

Dark blue colonies from the HiCrome *Cronobacter* spp. agar were purified by streaking onto tryptone soy agar (TSA) (Oxoid CM0131) and incubating at 22°C for 24-48 h^13^^)^. Cultures were subjected to Gram staining, oxidase test (Sigma-Aldrich 40560) and catalase test. Biochemical identification was carried out using the Remel RapID ONE System (Thermo Fisher R8311006), according to manufacturer’s instructions.

### Genotypic Characterisation

DNA was extracted from bacterial isolates using InstaGene Matrix (BioRad 7326030) according to manufacturer’s instructions. Extracted DNA samples were used in polymerase chain reactions (PCR). Genotypic characterisation of isolates was by 16S-23S rDNA internal transcribed spacer region PCR (ITS-PCR). ITS-PCR was carried out as described by Polit et al*.*^16^^)^.

ITS-PCR products were visualized by electrophoresis on 1.2% (w/v) agarose gels (Bioline BIO-41025). Gels contained 2 µl GelRed stain (41003, Biotium, Fremont, CA, USA). 10 µl of PCR product was mixed with 2 µl of loading dye (R0611, Thermo Fisher Scientific, Loughborough, UK) and loaded into the wells. A DNA molecular size marker (SM1113, Thermo Fisher Scientific) was used to estimate the sizes of the PCR products. The gels were run in a 1x Tris-Borate-EDTA (TBE) buffer at 60 V for approximately 1 hour. Images of the gels were taken using a UV transilluminator (Gel Doc EZ Imager, BioRad).

## Results and Discussion

It appeared that insect samples that originated from the Netherlands were processed by freeze-drying, whereas those originating from Thailand and Colombia were possibly processed by dehydration methods other than freeze-drying (Table 1). This may reflect the fact that freeze-drying is more expensive^17^^)^, and as such, probably more affordable in more developed countries such as the Netherlands.

Total aerobic counts from the ready-to-eat insects ranged from 2.00 log CFU/g (silkworm pupae) to 4.59 log CFU/g (buffalo worms) (Table 1). No *Enterobacteriaceae* were recovered from any of the whole insect samples (Table 1). This may be the result of blanching in boiling water for a short time, which usually occurs prior to drying of edible insects^3^^,^^18^^)^. Only the cricket flour showed any growth of *Enterobacteriaceae* on the VRBG agar. This could be because, unlike the other insect samples, the cricket flour is finely milled, which means that the crickets’ intestinal microbiota are distributed throughout the product^3^^)^. Nevertheless, all the samples could be considered as having low microbial load and meeting recommended hygiene criteria^4^^,^^19^^)^.

Out of the nine insect samples, five yielded positive results for both the CSB and HiCrome *Cronobacter* spp. agar. All five isolates were phenotypically characterized as gram negative, oxidase negative, catalase positive rods, forming yellow colonies on TSA at 22°C. Of these five samples that gave positive results in CSB after pre-enrichment, four gave no growth on VRBG agar, suggesting the presumptive *Cronobacter* spp. may have been stressed^6^^)^. Stressed or injured cells may not grow on selective media and require recovery via an enrichment step^20^^)^. This demonstrates that culture-based methods of hygiene determination may give misleading results. Therefore molecular methods are recommended, although they do not give an indication of the viability of the organisms detected, and should be used in combination with culture-based methods^18^^)^.

The Remel RapID ONE kit identified both reference strains, and four isolates (buffalo worm, cricket flour, giant waterbug, queen leafcutter ant) as *C. sakazakii*. The mealworm isolate was identified as *Pantoea agglomerans* (Table 2). Non-*Cronobacter* spp. would generally be differentiated on selective chromogenic agar^20^^,^^21^^)^. This therefore suggests misidentification of the mealworm isolate.

**Table 2.** Biochemical identification of reference strains and insect isolates

**Sample**	**Remel RapID ONE identification** **(% probability)**
NCIMB 8272	*Cronobacter sakazakii* (99.9%)
NCIMB 5920	*Cronobacter sakazakii* (99.9%)
Buffalo Worms	*Cronobacter sakazakii* (96.25%)
Cricket Flour	*Cronobacter sakazakii* (99.9%)
Giant Waterbug	*Cronobacter sakazakii* (99.9%)
Mealworms	*Pantoea agglomerans* (99.9%)
Queen Leafcutter Ants	*Cronobacter sakazakii* (99.9%)

**Fig. 1.**
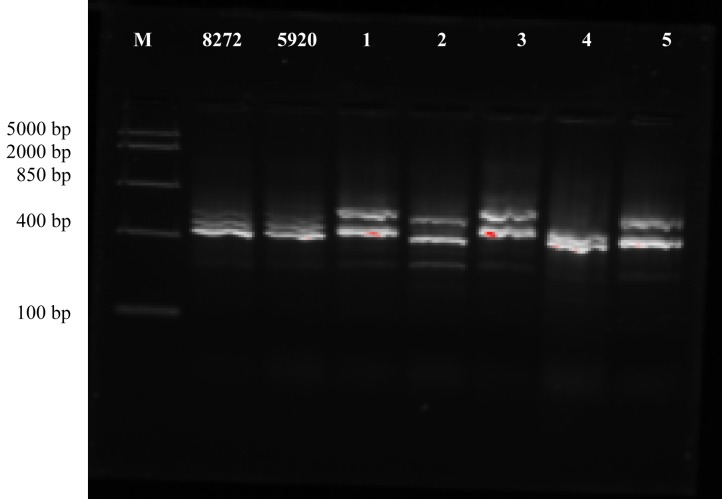
ITS-PCR band profiles of reference strains NCIMB 8272, NCIMB 5920 and insect isolates. M, marker; 1, mealworm; 2, buffalo worm; 3, queen leafcutter ant; 4, cricket flour; 5, giant waterbug.

DNA sequence-based methods, such as 16S rDNA sequencing, are considered to be the most reliable for identifying and confirming *Cronobacter* spp.^22^^)^. However, they are not always feasible, due to factors such as cost and time^22^^,^^23^^)^. Other PCR-based methods are therefore an alternative. ITS-PCR allows discrimination at species level, due to variations in the ITS region within a genus^24^^)^. Results of the ITS-PCR (Fig. 1) showed that both *C. sakazakii* reference strains had similar band patterns. None of the isolates exhibited similar band patterns as the *C. sakazakii* reference strains, suggesting they might not be *C. sakazakii*, but instead other species of *Cronobacter.* In addition, the mealworm, queen leafcutter ant and giant waterbug isolates appeared to have a similar genetic identity.

The five isolates may therefore be other species of *Cronobacter*, but this did not correspond with the Remel RapID ONE identification, further suggesting misidentification of the isolates. Misidentification by the Remel RapID ONE system is a possibility because the subjective nature of the results of such biochemical kits makes them limited in their reliability^25^^,^^26^^)^. Furthermore, the Remel RapID ONE system only has *C. sakazakii* on its database, meaning that other *Cronobacter* species cannot be reliably identified.

Presumptive *Cronobacter* spp. were detected in samples of ready-to-eat insects. Nevertheless, the risk of infection could be considered low for healthy individuals. Immunocompromised persons and the elderly may need to exercise caution when consuming ready-to-eat edible insects. ITS-PCR was useful for genotypic characterisation of the isolates. However, reference strains of other *Cronobacter* species should be included for effective identification of isolates. More studies that include a wider range of products from different companies are recommended.
